# E-SAP: Efficient-Strong Authentication Protocol for Healthcare Applications Using Wireless Medical Sensor Networks

**DOI:** 10.3390/s120201625

**Published:** 2012-02-07

**Authors:** Pardeep Kumar, Sang-Gon Lee, Hoon-Jae Lee

**Affiliations:** 1 Department of Ubiquitous-IT, Graduate School of Design & IT, Dongseo University, Sasang-Gu, Busan 617-716, Korea; E-Mail: pradeepkhl@gmail.com; 2 Division of Computer & Information Engineering, Dongseo University, San 69-1, Jurye-2-Dong, Sasang-Gu, Busan 617-716, Korea; E-Mail: nok60@dongseo.ac.kr (S.-G.L.)

**Keywords:** medical sensor network, secure healthcare, user authentication, mutual authentication, session key establishment, smart card

## Abstract

A wireless medical sensor network (WMSN) can sense humans’ physiological signs without sacrificing patient comfort and transmit patient vital signs to health professionals’ hand-held devices. The patient physiological data are highly sensitive and WMSNs are extremely vulnerable to many attacks. Therefore, it must be ensured that patients’ medical signs are not exposed to unauthorized users. Consequently, strong user authentication is the main concern for the success and large scale deployment of WMSNs. In this regard, this paper presents an efficient, strong authentication protocol, named E-SAP, for healthcare application using WMSNs. The proposed E-SAP includes: (1) a two-factor (*i.e.*, password and smartcard) professional authentication; (2) mutual authentication between the professional and the medical sensor; (3) symmetric encryption/decryption for providing message confidentiality; (4) establishment of a secure session key at the end of authentication; and (5) professionals can change their password. Further, the proposed protocol requires three message exchanges between the professional, medical sensor node and gateway node, and achieves efficiency (*i.e.*, low computation and communication cost). Through the formal analysis, security analysis and performance analysis, we demonstrate that E-SAP is more secure against many practical attacks, and allows a tradeoff between the security and the performance cost for healthcare application using WMSNs.

## 1. Introduction

During the last few years, we have seen the great emergence of wireless medical sensor networks (WMSNs) in the healthcare industry. Wireless medical sensors are the cutting edge components for healthcare application and provide drastically improved quality-of-care without sacrificing patient comfort.

**Figure 1.**
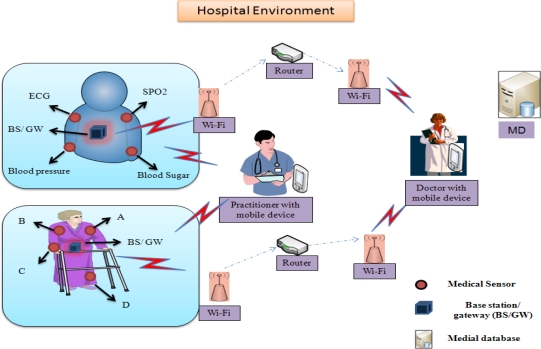
Patient monitoring using a wireless medical sensor network in a hospital environment.

A wireless medical sensor network is a network that consists of lightweight devices with limited memory, low computation processing, low-battery power and low bandwidth [1]. These medical sensors (e.g., ECG electrodes, pulse oxi-meter, blood pressure, and temperature sensors) are deployed on patient’s body and collect the individual’s physiological data and sends the collected data via a wireless channel to health professionals’ hand-held devices (*i.e.*, PDA, iPhone, laptop, *etc.*). A physician can use these medical sensor readings to gain a broader assessment of patient’s health status. The patient’s physiological data may include heartbeat rates, temperature, blood pressure, blood oxygen level, *etc.* A typical patient monitoring in hospital environment is shown in Figure 1.

Several research groups and projects are working in health monitoring using wireless sensor networks, for example, CodeBlue [2], LiveNet [3], MobiHealth [4], UbiMon [5], Alarm-Net [6], ReMoteCare [7], SPINE [8], *etc.* Thus, healthcare systems are the applications that most benefit from using wireless medical sensor technology that can perform patient care within hospitals, clinics and homecare.

Wireless medical sensor technology has offered tremendous advantages to healthcare applications, such as continuous patient monitoring, mass-causality disaster monitoring, large-scale in-field medical monitoring, emergency response, *etc.* Further, these WMSNs provide many new ways for acute disease analysis (e.g., motion analysis for Parkinson’s disease) [9,10].

However, wireless healthcare development has many challenges, such as reliable data transmission, fast event detection, timely delivery of data, power management, node computation and middleware [8,11–17]. Further, patients’ security and privacy is one of the big concerns for healthcare applications, especially when it comes to adopting a wireless healthcare system (*i.e.*, wireless medical sensors, wireless gateways, mobile devices, *etc.* [18]). Although wireless healthcare offers many advantages to patient monitoring, the physiological data of an individual are highly vulnerable. Further, due to the wireless nature of devices (*i.e.*, medical sensors, iPhone, PDA, *etc.*), the patients’ vital signs are much easier to query and monitor (*i.e.*, in an *ad hoc* manner) within the hospital ward rooms using smart phones, iPhones, PDAs, and laptops, so any adversary can be eavesdropping on patients locally in the ward room using their hand-devices that could cause of patient privacy breaches. More importantly, the patient vitals are very sensitive; so they (*i.e.*, the patient’s vitals) must be kept secure from unauthorized users and security threats [19–28]. Moreover, government laws (e.g., the Health Insurance Portability and Accountability Act of 1996 (HIPAA)) also regulated stringent rules for healthcare providers, such as; individuals’ vital signs are only revealed to authorized professionals (*i.e.*, doctors, caregivers and nurses) and family members [29,30]. A healthcare provider is subject to strict civil and criminal penalties (*i.e.*, either fine or imprisonment) if HIPAA rules are not followed properly [29,30]. Furthermore, as wireless medical sensor nodes themselves provide services to users (doctors, nurses, and technicians, are a few examples) it is necessary to control who is accessing their (the medical sensors’) information and whether they are authenticated to do so. Therefore, strong user authentication is a core requirement to protect from illegal access to patients’ vital signs, and can attain the highest levels of patients’ privacy.

So far many significant researches have been proposed for healthcare using sensor networks and provide sufficient security, such as data confidentiality, authentication, integrity and preserving patient privacy [31–39]. These schemes do not considere strong user authentication, and hence, lack a security mechanism, according to the HIPAA laws [29,30]. Further, in [40–46] the authors proposed a few user authentication protocol for wireless sensor networks, which are either broken or provide less security at very high computation and communication costs. Consequently, to the best of our knowledge, a strong user authentication (*i.e.*, professional authentication) protocol for wireless healthcare applications has not yet been addressed effectively in order to prevent illegal access to wireless medical sensor data.

In this paper, we discuss: (1) the healthcare architecture and major security requirements for healthcare application using wireless medical sensor networks; and (2) propose an efficient-strong authentication protocol, named E-SAP, for healthcare applications using WMSNs. The proposed scheme uses two-factor (*i.e.*, password and smartcard) user authentication, where each user must prove their authenticity first and then access the patient vital signs. (Note: We used user and professional, interchangeably and user or professional may be a doctor, a nurse, a surgeon or a technician. Furthermore, it is now widely believed that two-factor authentication provides strong and high level of security (*i.e.*, secure access of individual physiological data from wireless sensors) [29,30,47]).

In addition, E-SAP provides secure session key establishment between the users and the medical sensor nodes, and allow users to change their password. Furthermore, we demonstrate the formal verification of the proposed protocol by the Burrows, Abadi and Needham (BAN) logic model [48], where two main security properties are verified: authenticity and secure session key establishment. Moreover, the proposed scheme resists many practical attacks (e.g., replay, user and gateway masquerade, smartcard stolen-verifier, gateway secret key guessing, password guessing, and information-leakage). To attain the low computational overheads, our scheme uses one-way hash functions along with XOR operations and symmetric cryptosystem.

The rest of paper is organized as follows: Section 2 discusses the healthcare architecture using wireless medical sensors, adversary attack model, and wireless healthcare security requirements. Section 3 briefly reviews the related literature for secure healthcare monitoring using medical sensor networks. Section 4 introduces and describes a novel E-SAP: efficient-strong authentication protocol for healthcare application using WMSNs. Section 5 describes the brief introduction of BAN logic and provides formal verification of E-SAP using the BAN logic model. Section 6 discusses the security analysis and efficiency evaluation in contrast to exiting schemes and finally, in Section 7 conclusions and future directions are presented.

## 2. Healthcare Architecture, Adversary Attack Model, and Security Requirements for Healthcare Application in WMSN

This section presents healthcare monitoring architecture for hospital environments, adversary attack models and security requirements for healthcare application using WMSNs.

### 2.1. Healthcare Architecture

**Figure 2.**
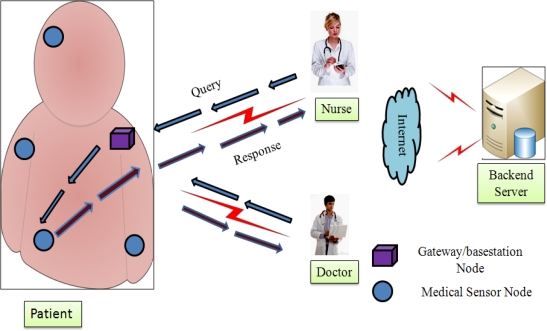
Healthcare architecture for patient monitoring.

A patient healthcare monitoring architecture is depicted in Figure 2, where usual patient monitoring is needed after patient hospitalization (e.g., after cardiac infarction). When a patient is hospitalized, he/she can get some suitable medical sensor devices, deployed strategically on the patient’s body. These sensors sense the health parameters, (e.g., blood pressure, movement, breathing, ECG *etc.*) and send physiological parameters to the professionals’ mobile devices (such as PDA, smart phone and laptop).

Later, a professional may store patient data on the backend server for further processing, which is currently outside the scope of this paper. It is obvious that a professional can access the patient’s health parameters directly from the medical sensor, in an *ad-hoc* manner.

As shown in the Figure 2, the healthcare architecture has three active entities, namely, user, medical sensors and base-station/gateway. We assume a real-time scenario, and suppose a professional wants to query the patient’s medical sensors for physiological information, as follows: (a) the user *(U_i_)* sends a query to the gateway node *(GW)*; (b) upon receiving the professional’s request, the gateway node forwards the user’s query to the medical sensor; and (c) thereafter, the medical sensor responds to the user. Here, the gateway node plays an important role between the professional and the medical sensor. Based on the above scenario, the next sub-section describes an adversary attack model for healthcare application using WMSNs.

### 2.2. Adversary Attack Model

The patient’s physiological information is very sensitive and may attract many attackers, such as insurance companies, corrupt media persons, individual enemies, *etc.* Furthermore, the patient’s medical sensors and the professionals’ hand-held devices are wireless in nature. So, these wireless devices may attract unauthorized users or thieves, more especially. For example, they (unauthorized users or thieves) can roam to the hospital ward and easily eavesdrop on the patients locally, so we have categorized the attack models as follows:

#### 2.2.1. Eavesdropping on Wireless Medical Data

As the medical sensors sense the patient’s body data, they transmit it over the radio communication channel. The wireless transmission ranges are not confined to hospital wards and these wireless channels are highly susceptible. As a result, an attacker may eavesdrop air messages (*i.e.*, a patient’s physiological information), and can disclose the patient’s physiological information. Hence, the patient privacy is breached.

#### 2.2.2. Active Attack

In an active attack scenario, the capability of an attacker depends on his/her skill (*i.e.*, ability to monitor all the communication). An attacker may inject bogus messages into the wireless channel and may alter the wireless medical sensor data during the communication. Any spurious messages injection into the healthcare network could cause mistreatment. Furthermore, an attacker may replay the old messages again and again, which could cause overtreatment (*i.e.*, medicine overdose). Thus, active attacks endanger and may pose a life-threatening risk to the patients.

### 2.3. Security Requirements for Healthcare Application Using Wireless Medical Sensor Networks

Based on the above attack model and literature survey [19–28] and [31–39], this sub-section sketches out the paramount security requirements for healthcare application in WMSNs, as follows:

#### 2.3.1. Strong User Authentication

The major problem in wireless healthcare environments is the vulnerability of wireless messages to access by unauthorized users, so it is desirable that strong user authentication be considered, where each user must prove their authenticity before accessing the patient’s physiological information. Furthermore, strong user authentication, also known as two-factor authentication, provides greater security for healthcare application using wireless medical sensor networks [47].

#### 2.3.2. Mutual Authentication

In real-time healthcare applications, the user and the medical sensor must authenticate each other; hence, they can ensure the communication is established between the authenticated user and the medical sensors.

#### 2.3.3. Confidentiality

The patient health data are highly sensitive and medical sensors are wireless in nature, therefore patient physiological data should remain confidential from passive attacks such as eavesdropping or traffic analysis. Thus, patient’s health data is only accessed or used by authorized professionals.

#### 2.3.4. Session Key Establishment

A session key should be established between a user/professional and a medical sensor node, so that subsequent communication could take place securely.

#### 2.3.5. Low Communication and Computational Cost

Since wireless medical sensors are resource constrained devices, and the healthcare application’s functions also need room for executing their tasks, the protocol must be efficient in terms of communication and computational cost.

#### 2.3.6. Data Freshness

Generally, professionals need patient physiological data at regular intervals, so there must be guarantee that patient health data is recent or fresh. Furthermore, it (data freshness) also ensures that an adversary cannot replay the old messages.

#### 2.3.7. Secure Against Popular Attacks

In real-time healthcare environments the protocol should be defensive against different popular attacks, such as replay attack, impersonation attack, stolen-verifier attack, password guessing attack, and information-leakage attack. As a result, the protocol can be easily applicable to the real-time wireless healthcare applications.

#### 2.3.8. User-Friendliness

The healthcare architecture should be easy to deploy as well as user-friendly; such as, a user can update his/her password securely, whenever he/she needs to.

## 3. Related Work

This section discusses the literature reviewed for secure healthcare monitoring using wireless sensor networks and general user authentication protocols for wireless sensor networks that have been recently proposed.

Malasri *et al.* [31] designed and implemented a secure wireless mote-base medical sensor network for healthcare applications. The main components of their scheme are: (i) two-tier architecture is designed for the patient data authentication; (ii) a secure key exchange protocol (*i.e.*, elliptic curve cryptography (ECC)) is used to establish secret shared keys between the sensor nodes and the base station; and (iii) a symmetric encryption/decryption algorithm provides confidentiality and integrity to patient data. Moreover, in their architecture each sensor mote has incorporated a fingerprint scanner; by doing so, the patient’s identity is verified with the aid of a base station. Although, their scheme provides adequate security to patients, it does not care about the strong professional authentication (*i.e.*, who is accessing the patients’ vital signs), whereas user authentication is a prime concern under various laws [29].

Hu *et al.* [32] have designed and proposed a software and hardware based real-time cardiac patient healthcare monitoring system named ‘tele-cardiology sensor network’ (TSN). TSN is particularly intended for the U.S. healthcare society. It enables real-time healthcare data collection for elderly patients in a large nursing home. In this architecture, a patient’s ECG signals are automatically collected and processed by an ECG sensor and transmitted in a timely way through a wireless channel to an ECG server for further analysis. TSN integrates with large number of wireless ECG communication units; each unit being called a mobile platform. A block cipher algorithm (*i.e.*, skipjack) is used for securing ECG data transmission, and protecting patient privacy. Although their proposal provides privacy in term of confidentiality and achieves integrity, strong user authentication is not addressed effectively.

Huang *et al.* [18] proposed a secure hierarchical sensor-based healthcare monitoring architecture. The proposed architecture has three network tiers (*i.e.*, sensor network, mobile network, and back-end network), and has considered three real-time healthcare applications (*i.e.*, in-hospital, in-home, and nursing-house) scenarios. The authors used wearable sensor systems (WSS) and wireless sensor motes (WSM) at the sensor network tier. The WSS are Bluetooth enabled and integrated with biomedical sensors; and the WSS are strategically placed on the patient’s body, whereas, the WSMs are deployed within the building, and are used to collect the environmental parameters and transmit through the Zig-bee wireless network standard. WSS and WSM broadcast data securely to the upper layer. Here, WSS uses an advance encryption standard (AES)-based authentication and encryption, while WSM uses a polynomial-based encryption scheme to establish secure point-to-point communication between two WSMs. In the mobile network tier, mobile computing devices (MCDs) such as PDAs are organized as an *ad-hoc* network and connected to the local station. MCD has the more computational capabilities to analyze the WSS and WSM data. The back-end tier is structured with a fixed station as a server, that provides application level services for lower tiers and process various sensed data from MCDs. Even though Huang *et al.* proposed a secure pervasive hierarchical sensor-based healthcare monitoring, they did not consider the need for strong user authentication, which is an imperative security for healthcare applications according to laws (*i.e.*, HIPAA [29]).

Very recently, Le *et al.* [34] suggested a mutual authentication and access control protocol (MAACE) where legitimate professionals can access their patient’s data. The MAACE facilitates mutual authentication and access control, which is based on elliptic curve cryptography (ECC). Furthermore, these authors argue that their scheme is secure enough in practical attacks, *e.g.*, replay attack, and denial-of-service attacks. Their architecture (*i.e.*, MAACE) consists of three layers: (i) sensor network layer (SN); (ii) coordination network layer (CN); and (iii) data access layer (DA). In their architecture, the SN transmits data to the CN (*i.e.*, PDA, laptop or cell phone), later, the data is forwarded to the DA for future record. Although, Le *et al.*’s protocol facilitates sufficient security against practical attacks, but their scheme susceptible to information-leakage attacks, which could be risky for the patient’s privacy. As a result, patient vital signs are could exposed to illegal users (e.g., insurance agents, media persons, *etc.*), which is not acceptable for real-time healthcare applications. Thus, a strong user authentication is required for the healthcare application using sensor networks.

In 2009, Das [42] has proposed two-factor user authentication protocol for wireless sensor networks. Das claimed that his protocol is safe against many attacks (*i.e.*, replay attack, password-guessing attack, user impersonation attack, node compromise attack, and stolen-verifier attack). Later, others [44,46] have pointed out that Das protocol is susceptible to the gateway bypass attack, user impersonation attack, insider attack, *etc*. Furthermore, Das’ protocol does not provide message confidentiality, and mutual authentication between the sensor and the user. Consequently, this protocol is not applicable to healthcare applications using sensor networks.

In [49], Kumar-Lee has shown that some authentication protocols [44,46] have security weaknesses and the computation costs of their protocols are very expensive. Thus, the protocols in [44] and [46] are not suitable for such wireless healthcare applications.

As we can notice from the above literatures, strong user authentication for healthcare application using wireless medical sensor networks has not yet been addressed adequately. Hence, a significant research effort is still required to explore the user authentication for WSN healthcare application. So, next section proposes an efficient-strong authentication protocol, named E-SAP, for healthcare applications using WMSNs.

## 4. The Proposed E-SAP Protocol

This section presents the proposed efficient-strong authentication protocol (E-SAP) where only legitimate professionals can access the patient’s body data in an authentic manner. The proposed protocol can be applicable to hospitals, homes and clinical environments. The basic idea of E-SAP is quite simple: professionals need to register with the gateway node at hospital registration center. Upon successful registration, the professional receives a smart card from the registration center. Then, professionals can access the patient physiological information’s from the patient body area sensor network, whenever demanded. In order to prove the professional legitimacy, a professional sends his/her password and smart card based login request to the gateway node. Upon receiving the professional requests the gateway node first authenticates him/her, and then forwards the professional’s request to the dedicated medical sensor, whose data the user is demanding. Thereafter, the medical sensor checks the authenticity of the gateway node and establishes a secure session key between the medical sensor and the professional and responds to the professional. In order to execute the proposed protocol, we have considered the following assumptions:

1. We assumed that the hospital registration center is a trusted authority.
2. The gateway node has three long master keys (*i.e.*, *J*, *K and Q* (256 bits long each)).
3. Initially, it is assumed that the gateway and the medical sensor nodes share a long-term secret key *SK_gs_* *= h(Q||ID_g_)* using any key agreement method [50,51].

Table 1 gives a list of notations with descriptions which are used throughout in the paper.

The proposed E-SAP consists of four phases, namely, the professional registration phase, patient registration phase, login and authentication phase, and password change phase.

### 4.1. Professional Registration Phase

In this phase, the professional initially needs to register with the gateway node at the registration center, as follows:

- User chooses *ID_i_* and *PW_i_* and submits to *GW* node using secure channel.
- Upon receiving user’s *ID_i_* and *PW_i_*, the *GW* node computes the following:
  - *C_ig_* *= E_J_[ID_i_||ID_g_]*
  - N_i_= h(ID_i_⊕PW_i_⊕K)

Thereafter, the *GW* node issues a smart card to the professional with the following *{h(.)*, *C_ig_*, *N_i_*, *K}*. Here, *K* is a long-term *GW* node secret, which is securely stored in the smart card.

### 4.2. Patient Registration Phase

In order to execute the proposed E-SAP, a patient needs to register at the hospital registration center [38], as follows:

- Patient passes his/her name to the registration center.
- After patient registration, registration center choose the suitable sensor kit (*i.e.*, medical sensor and gateway) and designate professionals/users.
- Later, registration center sends patient *ID_pt_* and medical sensors kit information (*i.e.*, gateway, sensor *etc.*) to the designated professionals/users.

Now, the technician deploys wireless medical sensors on the patient body area, strategically, as shown in Figure 2.

### 4.3. Login and Authentication Phase

This phase is invoked when a professional roams into the patients’ ward and wants to perform a query or to access the patients’ physiological information from the body network. This phase is further divided into login phase and authentication phase.

#### 4.3.1. Login Phase

The professional inserts his/her smart card into the terminal and inputs keys, *ID_i_* and *PW_i_*. Upon receiving the login request, the smart card verifies the user locally with pre-stored values and performs operations, as follows:

- N_i_* = *h(ID_i_⊕PW_i_⊕K)*and compare N_i_* = *N_i_,* if yes, then go to the next step, otherwise, terminates the request.
- Compute: *h(ID_i_)* and *CID_i_* = *E_K_[h(ID_i_)||M||Sn||C_ig_||T*′*]*. Here, *M* is a random nonce that is generated by professional system, which is used to establish the secure session key.

Then professional’s system sends message *<CID_i_*, *T*′*>* to *GW* node. Here, *T*′ is the current time stamp of professional system.

#### 4.3.2. Authentication Phase

This phase is invoked when the *GW* node receives a login request from a professional. Upon receiving the login request at time *T*″, the *GW* node performs the following and authenticates him/her, as:

- Validate the time *T:* check*, if (T*″ − *T*′*) ≥ ΔT*, if yes, then rejects the request and aborts any further process. Otherwise, it performs the next steps. Here, *T*″ is the current time of *GW* node and *ΔT* is the time interval for the transmission delay.
- Decrypt sub-message *CID_i_* using key *K (i.e.*, *D_K_ [CID_i_])* and obtain *h(ID_i_)^§^*, *Sn*, *M* and *T*′*^§^*. Similarly, decrypt sub-message *Cig* using the shared key *J* (*i.e.*, *D_J_ [C_ig_])* and obtain ID_i_* and ID_g_*.
- Compute h(ID_i_)*, and compare h(ID_i_)* = h(ID_i_)^§^, ID_g_* = *ID_g_* and *T*′ = *T*′*^§^*, if yes, then the request is authentic; otherwise, terminate any further processes.
- Compute: *A_i_* = *E_SKgs_ [ID_i_||Sn||M||T*′″*||T*′*]*, here *T*′″ is the current time stamp of *GW* node. Thereafter, the *GW* node sends a message *<A_i_, T*′″*>* to the medical sensor that the professional wants to access. Furthermore, *A_i_* ensures to the medical sensor that the request has come from the legal gateway node.

Upon receiving the gateway node message, the medical sensor node performs the following steps:

- Validate the time *T*: check, if *(T*″″ *− T*″′*) ≥ ΔT*, if yes, then it rejects the request and aborts any further process. Otherwise, it performs the next steps. Here, *T*″″ is the current time of the medical sensor node and *ΔT* is the time interval for the transmission delay.
- The medical sensor *(Sn)* decrypts the sub-message *A_i_* using shared key *SK_gs_ (i.e.*, *D_SKgs_ [A_i_])*, and obtains ID_i_*, Sn*, M*, T″′* and *T*′.
- Now, *Sn* compares Sn* = *Sn* and *T*″′ *= T*″′*, if not, then it aborts the request; otherwise it continues with the next steps.
- Compute session key *SK* = h (ID_i_*||Sn||M*||T′*)*, and message L = E_SK_ [Sn||M*||T*], here, T* is the current time stamp of the medical sensor node. After that, the medical sensor node sends a response message <L, T*> to the professional.

Upon receiving the medical sensor node response, the professional validates the time as follows:

- Validate the time T*: check, if (T** − T*) ≥ ΔT, if yes, then it rejects the request and terminates. Otherwise, it continues with the further process. Here, T** is the current time of the professional system and *ΔT* is the time interval for the transmission delay.
- The professional system computes *SK* = *h (ID_i_||Sn||M||T*′*)*.
- Decrypt the message *L* using *SK*, and obtain Sn* and M*. Thereafter, compare Sn*= Sn, and M* = *M*, if yes, then a secure session key has been established; otherwise not.

**Figure 3.**
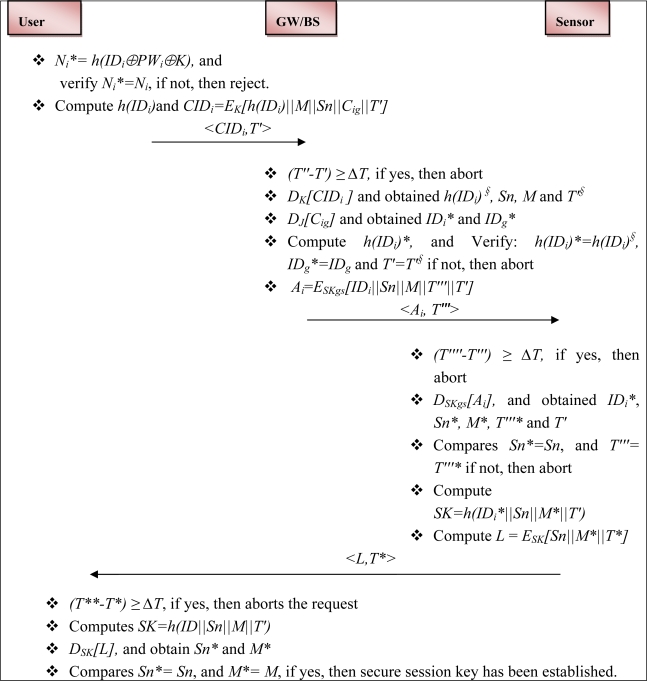
Flow of the Login and Authentication phases.

The flow of the login and authentication phases is shown in Figure 3.

### 4.4. Password-Change Phase

The password-change phase is invoked when *U_i_* wants to change/update the password, when he/she requires. The password change procedure is as follows:

- The user inserts his/her smart card into the terminal and enter keys *(i.e.*, *ID_i_ and PW_i_)*.
- Smart card performs the operations:
  - N_i_* = h(ID_i_⊕PW_i_⊕K)
  - Compare N_i_* = N_i_, if yes, then perform the next step; otherwise abort the operation.
- Enter new password *PW_inew_*.
- Compute *N_inew_* = *h (ID_i_⊕PW_inew_⊕K)*.
- Replace *N_i_* with *N_inew_* from the smart card.

## 5. Formal Analysis of E-SAP Using BAN Logic

Formal analysis ensures that the protocol functions are correctly modeled, and needs to be verified, (*i.e.*, error free) before their real-time implementation [48]. In this regards, this section describes the formal verification of E-SAP using BAN logic, which is popular for formal verification of authentication protocols. The section is divided into: (A) brief overview of the BAN logic, which was introduced by Burrows, Abadi and Needham [48]; and (B) a demonstration of the formal execution and validity proofs of the proposed E-SAP using the BAN authentication logic model.

### 5.1. BAN Logic

The BAN logic is a popular authentication protocols analysis model, and it is useful to prove the validity of authentication and key establishment protocols, for more details the readers may refer to [48]. The notations used in BAN logic are defined as follows:

- *P believes X*: The main construct of logic is ‘*P believes X*’ (*i.e.*, *the principal P believes on X*) or *P* would be entitled to believe *X*.
- *P sees X:* Only *‘P sees X’*, *i.e.*, suppose someone has sent a confidential message (*i.e.*, encrypted message) containing *X* to *P*, then *P* can read *X* (*i.e.*, after performing some decryption).
- *P said X:* The principal *‘P once said X’*; means, at some time the principal *P* sent a message including *X*.
- *P controls X:* The principal *‘P has controls over X’*; means, the principal *P* is an authority on *X* and should be trusted (e.g., a server is often assumed trusted and generate secret keys properly).
- *Fresh(X): Fresh(X)* means, *X* has not been sent recent in a message during the protocol execution. Furthermore, *Fresh(X)* protects from replay attack.
- $P\overset{K}{↔}Q$: The principal *P* and *Q* may use secret shared key *K* for secure communication. The keys *K* will never be disclosed to others except for the designated principals *(i.e.*, *P and Q)*.
- *{X}_K_*: Means the formula *X* is encrypted using the key *K.*
- <X>_Y_: The formula *X* is combined with secret parameter *Y*.

Now, we have defines some logical rules that we use in proofs, and which are directly adopted from [49], as follows:

- ➢ *Message-meaning rule*
  $$\frac{P\;\mathrm{believes}\;Q\overset{K}{↔}P,\;P\;\mathrm{sees}\;{\left\{X\right\}}_{K}}{P\;\mathrm{believes}\;Q\;\mathrm{said}\;X}$$
- ➢ *Nonce-verification rule*
  $$\frac{P\;\mathrm{believes}\;\mathrm{fresh}\;(X),\;\;P\;\mathrm{believes}\;Q\;\mathrm{said}\;X}{P\;\mathrm{believes}\;Q\;\mathrm{believes}\;X}$$
- ➢ *Controls rule*
  $$\frac{P\;\mathrm{believes}\;Q\;\mathrm{controls}\;X,\;P\;\mathrm{believes}\;Q\;\mathrm{believes}\;X}{P\;\mathrm{believes}\;X}$$
- ➢ *Fresh rule*
  $$\frac{P\;\mathrm{believes}\;\mathrm{fresh}\;(X)}{P\;\mathrm{believes}\;\mathrm{fresh}\;(X,Y)}$$

### 5.2. Formal Verification of the Proposed E-SAP

This sub-section demonstrates the formal verification of our proposed protocol using the BAN logic analysis model [48]. The main principals of E-SAP are: user (*U_i_)*, gateway *(GW)* and medical sensor node *(Sn)*. The following symbols are used: (a) the secret keys are *J*, *K*, *SK_gs_* and *SK*; (b) the time-stamps are *T*′, *T*″′ and T*. The main goal of formal verification is to establish a secure session key between the user and the medical sensor node. To perform the formal verification of E-SAP, we use the following logical postulates:

- *U_i_* ***believes*** $U_{i}\overset{\mathrm{SK}}{↔}\mathrm{Sn}$,
- *U_i_* ***believes*** *Sn* ***believes*** $U_{i}\overset{\mathrm{SK}}{↔}\mathrm{Sn}$
- *Sn* ***believes*** $U_{i}\overset{\mathrm{SK}}{↔}\mathrm{Sn}$,
- *Sn* ***believes*** *U_i_* ***believes*** $U_{i}\overset{\mathrm{SK}}{↔}\mathrm{Sn}$

The protocol messages (as shown in Figure 3) are needs to be transform into the idealized form, as shown in Table 2:

E-SAP formal analysis using BAN logic required further assumptions, as follows:

A1) *U_i_* ***believes*** $\mathrm{GW}\overset{K}{↔}U_{i}$
A2) *GW* ***believes*** $U_{i}\overset{K}{↔}\mathrm{GW}$
A3) *GW* ***believes*** $U_{i}\overset{J}{↔}\mathrm{GW}$
A4) *GW* ***believes*** $\mathrm{Sn}\overset{\mathrm{SKgs}}{↔}\mathrm{GW}$
A5) Sn believes $\mathrm{GW}\overset{\mathrm{SKgs}}{↔}\mathrm{Sn}$
A6) *Sn* ***believes*** $U_{i}\overset{\mathrm{SK}}{↔}\mathrm{Sn}$
A7) *U_i_* ***believes*** $\mathrm{Sn}\overset{\mathrm{SK}}{↔}U_{i}$
A8) *Sn* ***believes*** *(U_i_* ***controls*** $U_{i}\overset{\mathrm{SK}}{↔}\mathrm{Sn}$*)*
A9) *GW* ***believes*** $\mathrm{GW}\overset{J}{↔}\mathrm{GW}$
A10) *GW* ***believes*** *(U_i_* ***controls*** *ID_i_)*
A11) *U_i_* ***believes fresh*** *(M)*
A12) *U_i_* ***believes*** *Sn* ***fresh*** (T*)
A13) *GW* ***believes*** *U_i_* ***fresh*** *(T′)*
A14) *Sn* ***believes*** *GW* ***fresh*** *(T″′)*
A15) *Sn* ***believes*** *GW* ***fresh*** *(M)*
A16) *Sn* ***believes*** *(GW* ***controls*** *ID_i_)*

Based on the above assumptions and BAN logic rules, we perform the verification of the proposed E-SAP, as shown in Table 3.

As we can see from the above verification, *A7*, *S13*, *S19* and *S20* establish the secure session key between the user and the medical sensor. Furthermore, *A3*, *A10*, *S5*, *S11*, *S19* and *S20* verify mutual authentication between the user and medical sensor using the gateway. Hence, the goal of E-SAP is achieved (*i.e.*, secure session key has established and only authentic users can access an individual’s body information from the wireless medical sensor networks).

## 6. E-SAP Evaluation

This section discusses the security analysis and functionality analysis of the proposed E-SAP for healthcare application using medical sensor networks. Further, we present a performance analysis of E-SAP. The following assumptions are considered before evaluating the proposed protocol, which is based on a smart card and password (*i.e.*, two-factor):

- The adversary has total control of wireless communication; he/she may intercept, delete or alter any message in the communication (recall the discussion of attack model in Section 2).
- The attacker either obtains a user’s password, or extracts the secrets from the smart card through [52,53], but not both (*i.e.*, password and smart card) at the same time [50].
- Assumed that, extracting secrets from smart card is quite complex and some smart card manufacturer provide countermeasures against side channel attacks [42,50]. In [54] authors proposed some software countermeasures against power analysis attack.
- We assumed that the symmetric cryptosystems are secure enough to protect patient physiological information from cracking, and any encrypted text cannot be decrypted without having the secret keys, which is known only to the trusted entities (*i.e.*, user, gateway, medical sensor and hospital registration center).

### 6.1. Security Analysis

This sub-section shows the proposed protocol is secure against many practical attacks. In additions, the proposed E-SAP facilitates: confidentiality, mutual authentication between the user and the medical sensor, a secure session key establishment between the medical sensor node and the professionals, and professionals can change their password, securely.

***Replay attack***: The proposed protocol is resistant to replay attacks. Assume that an adversary replay the old captured messages to the gateway (*i.e.*, *<CID_i_,T*′*>)*, the medical sensor *(i.e.*, *<A_i_*, *T*″′*>)*, and the user *(i.e.*, <L,T*>). However, he/she (attacker) cannot pass the old messages, because all messages are validated by the fresh time stamps, which are contained in the protocol messages *(i.e.*, *(T*″*-T*′*) ≥ ΔT*, *(T*″″*-T*″′) ≥ ΔT and (T**-T*) ≥ ΔT.

***Masquerading user attack***: An attacker cannot masquerade as the professional *(U_i_)*. Suppose an adversary were able to forge a login message *<CID_i_, T*′*>*. Now the adversary will try to login into the WMSN with a modified message <CID_i_*, T′*>*. He/she cannot pass the fake message because the forged CID_i_* will not be verified at the gateway node and the gateway node cannot get the original message *(i.e.*, *(h(ID_i_)||Sn||M||C_ig_||T*′*))* by decrypting the fake CID_i_*.

***Masquerading gateway attack***: An attacker cannot impersonate a gateway, since he/she does not have any idea how to get *J*, *K* and *SK_gs_* from the protocol messages. So, masquerading as the gateway is not applicable to the E-SAP.

***Gateway secret guessing attack***: The proposed scheme is secure against the gateway secret guessing attack. The gateway has three master keys *(i.e.*, *J*, *K and Q)*, which are not transmitted as plaintext. Hence, E-SAP is secure against gateway secret guessing.

***Stolen verifier attack***: In [43], a user table (*i.e.*, *ID_i_ and PW_i_*) is stored on the gateway node, which may be a high risk to breach the security of protocols. In contrast, the E-SAP protocol does not use any *ID_i_* table and password table. So any stolen-verifier attack will not applicable on the proposed protocol.

***Password guessing attack***: An attacker cannot guess the password in our scheme. In the proposed protocol password is not passing as plaintext, instead *N_i_= h (ID_i_⊕PW_i_⊕K)*, so password guessing is not possible.

***Mutual authentication***: The proposed E-SAP provides mutual authentication between the user and the medical sensor. As shown in the Figure 3, the gateway sends message *<A_i_*, *T*″′*>* to the medical sensor. Here, *A_i_ = E_SKgs_ [ID_i_||Sn||M||T*″′*||T*′*]* and it ensures to the medical sensor that the message has come from the legitimate gateway node. Thus, the medical sensor believes that the user is a legitimate user. Furthermore, when the user receives a medical sensor message *<L*, T*>, then he/she verifies the medical sensor (*i.e.*, whether real or not). Hence, the proposed protocol achieves mutual authentication between the user and the medical sensor.

***Information-leakage attack***: The protocol information-leakage gives room to the attackers, which could be harmful for the patient privacy. In E-SAP, suppose an adversary eavesdrops the protocol messages *(i.e.*, *<CID_i_*, *T*′*> <A_i_*, *T*″′*> and <L*, T*>). Here, the sub-message *CID_i_* is encrypted using shared secret *K,* the message *A_i_* is encrypted using shared *SK_gs_*, and the sub-message *L* is encrypted using *SK.* Therefore, E-SAP messages information’s are not leaked during communication. As a result, information-leakage attacks not applicable to our protocol.

***Secure session key***: The proposed E-SAP establishes a secure session key between the user and the medical sensor node after the authentication phase taken place. As we can see in Figure 3, a session key (SK=h (ID_i_*||Sn||M*||T′*))* is setup between the medical sensor node and the user. Furthermore, the established session key provides confidentiality for subsequent communication; and for each session the session key will fresh.

***Confidentiality***: Confidentiality is a paramount requirement for healthcare application using wireless medical sensor networks. In the proposed E-SAP, the session key could be used for further secure subsequent communication between both (*i.e.*, user and medical sensor node may encrypt patient physiological information’s using the session key *(SK)*). Furthermore, the proposed protocol provides air message confidentiality to their messages *(CID_i_=E_K_ [h(ID_i_)||M||Sn|| C_ig_||T*′*]*, *A_i_=E_SKgs_ [ID_i_||Sn || M ||T*″′*||T*′]and L = E_SK_[Sn||M*||T*]).

***Secure password change***: In the password-change phase, the proposed protocol first verifies the user’s old password and identity, and only then requests a new password. Otherwise it rejects the password change request. Thus, the proposed scheme is secure against changed passwords.

### 6.2. E-SAP Functionality Analysis

This subsection shows the E-SAP functionality and makes a comparison with related schemes (*i.e.*, Le *et al.* [34], Das [42], Vaidya *et al.* [43] and He *et al.* [46]). As shown in Table 4, the proposed protocol provides more functionality such as strong user authentication, mutual authentication between the user and the medical sensor node, it establishes a secure session key for the user and the medical sensor node, message confidentiality and professionals are able to change their password, whereas in [34,42,43] and [46] the schemes provides less security functionality, which are paramount requirements (recall section 2-C) for wireless healthcare applications. Further, it can be seen from Table 4 that the proposed E-SAP is robust against many popular types of attacks (e.g., replay attack, masquerade attack, gateway secret guessing attack, and information-leakage attack) as compared to other schemes. It is worth notice that our protocol provides indispensable security features, whereas, the schemes in [34,42,43,46] provide less security functionality for real-time healthcare applications.

### 6.3. E-SAP Performance Evaluation

This subsection evaluates the performance of proposed protocol in term of computation cost, communication cost and compares the results with [34,42,43,46].

The performance evaluation parameters are:

T_pu_: public-key computation, T_pr_: private-key computation,

H (performing one hash function), S (symmetric-cryptosystem), and M (performing one message authentication code).

*Computation cost:* The medical sensor devices (*i.e.*, gateway node and sensor node) have limited power resources and computation capability. Therefore, the computation cost is a prime factor for resource constrained devices. The user registration computation cost is a one-time task and it is not a main concern, whereas the login and authentication computation cost are a prime concern due to the resource constrained nature of the gateway node and the medical sensors nodes. Table 5 shows the computation cost of the proposed E-SAP and related schemes, *i.e.*, Das [42], Vaidya *et al.* [43], and He *et al.* [46]. It is easy to see from Table 5, in registration phase the proposed E-SAP needs only 1H and 1S at GW node, whereas [42,43,46] require, 3H, 4H and 5H, respectively, which a is high computation cost at GW node.

Further, the Le *et al.* [34] scheme requires modular exponentiation to compute the public and private keys, so their scheme is computationally expensive and time-consuming, and it also needs to generate and verify digital certificates. In the login and authentication phase, E-SAP requires 6H and 7S, and provides more security. In contrast [34,42,43,46] require 4H+4S+6M, 7H, 9H and 7H, respectively, and provide less security services. This is due to the fact that the proposed E-SAP incurred more computation cost and provides paramount security functionality to healthcare applications as compared to [42,43,46]. Thus, the computation cost of E-SAP is well-suited to the healthcare applications using wireless medical sensor networks.

*Communication cost:* The communication cost is an important issue in wireless communication, (*i.e.*, more message exchanges consume more power). From Figure 3, it is easy to visualize that the proposed E-SAP requires three message exchanges between the user, the gateway and the medical sensor, whereas the schemes in [42] and [46] require three message exchanges, and [34] and [43] require four exchanges. Hence, the proposed protocol is well-suited and quite simple in enhancing the wireless communication security for healthcare application.

Considering the functionality, computation cost, and communication cost of E-SAP, it is clear that our protocol is more efficient for healthcare applications using medical sensor networks as compared to others [34,42,43,46].

## 7. Conclusions

Wireless medical sensors offer services to professionals; but what do we do to verify the professionals (*i.e.*, authentic or not). That poses a question to researchers, how to protect medical sensor data from illegal users?

In order to solve the above questions, this paper proposed E-SAP, an efficient-strong user authentication protocol for healthcare application using wireless medical sensor networks. E-SAP utilizes two-factor security features and provides strong user authentication, confidentiality and session key establishment for healthcare application using WMSNs. It is noteworthy that E-SAP is more capable in terms of security services, computation and communication cost, as compared to other existing protocols. Furthermore, through intensive analysis (*i.e.*, BAN logic authentication model) we have shown that E-SAP achieves its stated security goals and is defensive against many popular types of attacks. It is a well-suited protocol for hospital, homecare, and clinic healthcare applications using wireless medical sensors.

The future directions for this study are: (1) to develop a real-time heterogeneous biomedical sensor network for healthcare monitoring, (2) implement E-SAP on a real-time test-bed for healthcare application, and (3) more focus on access control in patient mobility scenarios and strong patient privacy.

## Figures and Tables

**Table 1.** Notation and Description.

**Notations**	**Description**
*U_i_*	User *i^th^* want to login
*ID_i_*	*ID* of user *U_i_*
*PW_i_*	Password of user *U_i_*
*ID_pt_*	*Patient ID*
*GW*	Gateway node
*ID_g_*	Gateway *ID*
*Sn*	Sensor node
*J, K and Q*	Gateway secrets
*E_Key_[]*	Symmetric encryption using shared key.
*D_Key_[]*	Symmetric decryption using shared key.
*M*	User’s generated nonce
*h(.)*	One-way cryptographic hash function
*⊕*	XOR operation
*||*	Concatenation operation

**Table 2.** E-SAP messages transform into the idealized form.

*E-SAP messages:* Message 1:*U_i_→GW:<CID_i_,,T′>(i.e., E_K_[h(ID_i_)||M||Sn||C_ig_||T′], T′)* Message 2: *GW→ Sn: <A_i_,T″′>(i.e., E_SKgs_[ID_i_||Sn||M||T″′||T′], T″′)* Message 3: Sn→U_i_: <L, T*> (i.e., E_SK_[Sn||M*||T*], T*).

*Idealized form:* Message 1: *U_i_→GW:{h(ID_i_)||M||Sn||{ID_i_||ID_g_}_J_||T′}_K_, T′* Message 2: *GW→ Sn: {ID_i_||Sn||M||T″′||T′}_SKgs_, T″′* Message 3: Sn→U_i_: {Sn||M*||T*}_SK_, T* Session key SK = h(ID_i_*||Sn||M*||T′)

**Table 3.** Formal verification of E-SAP using BAN logic model.

Message 1: *U_i_→GW:{h(ID_i_)||M||Sn||{ID_i_||ID_g_}_J_||T*′*}_K_, T*′
*S1) GW* ***sees*** *{h(ID_i_)||M||Sn||{ID_i_||ID_g_}_J_||T′}_K_,T′ //* by *seeing rule* *S2) GW* ***believes*** $U_{i}\overset{K}{↔}\mathrm{GW}$ // by *A1, A2, S1, message-meaning rule* *S3) GW* ***believes*** *U_i_* ***controls*** *ID_i_ //* by *A10, controls rule* *S4) GW* ***believes*** *U_i_* ***fresh*** *(M) // by message-meaning and fresh rule* *S5) GW* ***believes*** $\mathrm{GW}\overset{J}{↔}\mathrm{GW}$*// by A9, S1, message-meaning rule* *S6) GW* ***believes*** *U_i_* ***fresh*** *(T′) // by S4, A13, fresh rule* *S7) GW* ***believes*** *Sn* ***said*** *{ID_i_||Sn||M||T″′||T′}_SKgs_, T″′ // by message-meaning rule*
Message 2: *GW→ Sn: {ID_i_||Sn||M||T*″′*||T*′*}_SKgs_, T*″′
*S8) Sn* **sees** *{ID_i_||Sn||M||T*″′*||T*′*}_SKgs_, T*″′ *// by seeing rule* *S9) Sn* ***believes*** *GW* ***fresh****(T″′) // by A14, fresh rule* *S10) Sn* ***believes*** $\mathrm{GW}\overset{\mathrm{SKg}s}{↔}\mathrm{Sn}$*// by A5, S8, message-meaning rule* *S11) Sn* ***believes*** *(GW* ***controls*** *ID_i_) // by S8, A16, controls rule* *S12) Sn* ***believes*** *GW* ***fresh****(M) // by A15, fresh rule* *S13) Sn* ***believes*** $U_{i}\overset{\mathrm{SK}}{↔}\mathrm{Sn}$*// by A6, S8, message-meaning rule* *S14 Sn* ***believes*** *U_i_* ***said*** {Sn||M*||T*}_SK_, T*
Message 3: Sn→U_i_: {Sn||M*||T*}SK, T*
*S15) U_i_****sees***{Sn||M*||T*}SK, T* // by seeing rule
*S16) U_i_****believes****Sn****fresh***(T*) // by A12, fresh rule
*S17) U_i_****believes***$\mathrm{Sn}\overset{\mathrm{SK}}{↔}U_{i}$*// by A7, S15, message-meaning rule*
*S18) U_i_****believes****Sn****fresh****(M) // by fresh rule*
*S19) U_i_****believes****Sn****believes***$U_{i}\overset{\mathrm{SK}}{↔}\mathrm{Sn}$*// by S 15, message-meaning rule*
*S20) U_i_****believes***$U_{i}\overset{\mathrm{SK}}{↔}\mathrm{Sn}$

**Table 4.** Comparison of E-SAP functionality with related schemes.

**Functionalities**	**[34]**	**[42]**	**[43]**	**[46]**	**Proposed E-SAP**
Strong user authentication	No	Yes	No	Yes	**Yes**
Mutual authentication between *U_i_* and *Sn*	Yes	No	Yes	No	**Yes**
Session key establishment	No	No	No	No	**Yes**
Secure password change	NA	No	Yes	Yes	**Yes**
Message confidentiality	No	No	No	No	**Yes**
Protection to replay message	Yes	Yes	Yes	Yes	**Yes**
Secure against *GW* secret key guessing attack	Yes	No	No	No	**Yes**
Secure against user masquerading attack	Yes	No	No	No	**Yes**
Secure against gateway masquerading attack	No	No	No	No	**Yes**
Secure against Information-leakage attack	No	No	No	No	**Yes**
Protocol formal verification	No	No	No	No	**Yes**

NA: Not applicable.

**Table 5.** Performance comparison of E-SAP with existing schemes.

**Schemes**	**Registration**		**Login and authentication**		

	**User**	**GW**	**User**	**GW**	**Sn**
Le *et al.*’s [32]	T_pu_+T_pr_	T_pr_	1H+1S+2M	2H+2S+2M	1H+1S+2M
Das’s [40]	−	3H	4H	4H	1H
Vaidya *et al.*’s[41]	2H	2H	3H	3H	3H
He *et al.*’s [44]	1H	5H	5H	5H	1H
**Proposed E-SAP**	**−**	**1H+1S**	**4H+2S**	**1H+3S**	**1H+2S**
